# Computational Chemistry and Toxicology of Phosphonate Esters of Alkyl Acetoacetates, an Unexplored Class of V-Agents

**DOI:** 10.3390/cimb48080779

**Published:** 2026-07-30

**Authors:** Georgios Pampalakis, Eleni Pontiki

**Affiliations:** 1 Laboratory of Pharmacology, School of Pharmacy, Aristotle University of Thessaloniki, 54124 Thessaloniki, Greece; 2 Laboratory of Pharmaceutical Chemistry, School of Pharmacy, Aristotle University of Thessaloniki, 54124 Thessaloniki, Greece; epontiki@pharm.auth.gr

**Keywords:** nerve agents, V-agents, vinyl phosphonates, chemical safety

## Abstract

V-agents are exceedingly toxic oily substances, among which phosphonothiolates VX and VR have been extensively studied. Nevertheless, V-agents encompass a large family of nerve agents with diverse structures, including the phosphonate esters of alkyl acetoacetates or 2-alkoxycarbonyl-1-methylvinyl cycloalkyl methylphosphonates. These agents exist in two geometric isomers, and their properties remain largely unknown. Due to continuous concerns about chemical terrorism and safety, it is necessary to study their properties in order to develop effective countermeasures. Here, we applied computational tools to predict their ADME profile, chemical properties, and structure-related toxicity. These agents exhibited optimal drug-like properties, and it was predicted that they can penetrate the skin, acting as percutaneous hazards, and further penetrate the gastrointestinal tract and the blood–brain barrier. Certain CYP450 enzymes could differentially recognize the E- and Z-isomers, and this may explain the observation that E-isomers are significantly more toxic than their Z-counterparts. Analyses of the E- and Z-isomer stabilities also offered evidence for the reported lability of the Z-isomers. In conclusion, this study provides the first detailed in silico description of these V-agents, requiring future targeted experimental validation.

## 1. Introduction

In the early 1950s, it was noted that vinyl phosphates have high insecticidal activity [1,2]. One of these compounds is the O,O-dimethyl 2-methoxycarbonyl-1-methylvinyl phosphate, also known as mevinphos (Phosdrin). A characteristic feature of this class of compounds is the presence of a double bond that gives rise to two different geometric isomers: the E- and Z-isomers. The commercial mevinphos product is a mixture of these isomers with an approximate composition of 67% E-isomer and 33% Z- isomer [1]. Mevinphos exhibits high toxicity, with an oral LD50 of 3.7 mg·kg^−1^ in rats [3].

Therefore, mevinphos has served as a lead compound for the development of exceedingly toxic chemical warfare agents. Specifically, its structure was modified by changing one O-alkoxy group to methylphosphonate to generate a new class of V-agents, as reported in an unclassified report [4]. This class of V-agents was also patented [5]. V-agents are among the most toxic of known nerve agents, along with the recently described A-agents (Novichoks) [6,7,8,9].

Mevinphos-like nerve agents constitute another class of V-agents with diverse structures that have remained almost unexplored in scientific literature [10]. Indeed, most experimental studies on V-agents have focused on VX and VR, as these two chemicals were stockpiled by the US and USSR respectively. Studies have particularly concentrated on chemistry, decontamination, toxicology, and medical countermeasures [11,12,13,14,15]. However, it is not known whether mevinphos-like nerve agents have been stockpiled. Importantly, EA 1671, the most toxic agent from this class, is solid, which may have hindered its weaponization relative to VX during the 1950–1960s.

However, due to the public availability of the methodology of preparation [4,5] and the concern of potential chemical terrorism, this class of agents should be investigated to understand its chemical and toxicological properties and to develop effective countermeasures. In addition, this class of V-agents is not included in the Chemical Weapons Convention (CWC) list of Schedule 1 chemicals (https://www.opcw.org/chemical-weapons-convention/annexes/annex-chemicals/schedule-1, assessed on 9 July 2026). This may further facilitate clandestine development.

Computational tools provide a rapid method for the assessment of the toxicological and chemical properties of new compounds and can also provide clues for future experimental directions. In addition to saving time and budget, computational studies reduce the risk of handling exceedingly toxic chemicals. Here, we applied computational pharmacology/toxicology and chemistry tools to predict the reactivity and potential biological behavior of the mevinphos V-type nerve agents. Previously, such methodologies were used to predict the properties of a new drug, even though only one Web-based program was applied [16]. In this study, to increase the validity of our results, we applied three different Web-based software products, and we also identified weaknesses of these programs that need to be improved in the future. We further used these tools to provide clues related to their chemical reactivity relative to VX (electrophilicity), as well as their ability to bind to acetylcholinesterase (AChE), the main target of nerve agents. The aim of the study is to provide an in silico characterization of their biochemical properties that can be validated in the future with targeted experiments.

## 2. Materials and Methods

### 2.1. Physicochemical Properties, Drug-likeness, and ADME

The prediction of physicochemical and drug-likeness (medicinal chemistry) properties, as well as the prediction of ADME, was carried out with SwissADME (https://www.swissadme.gr; accessed on 25 August 2025) [17], ADMETlab3.0 (https://admetlab3.scbdd.com; accessed on 25 August 2025) [18], and Deep-PK (https://biosig.lab.uq.edu.au/deeppk; accessed on 25 August 2025) [19]. All these programs are freely available and run predictions online. SwissADME and ADMETlab3.0 allow the design of a two-dimensional chemical formula of the compound under study or for it to be entered as a SMILES formula, while Deep-PK only allows the compound formula to be entered in SMILES format.

### 2.2. Online Docking

Docking was carried out with SwissDock, which is available on the SwissADME site—specifically, using the Docking with Attracting Cavities program [20]. The structure of human acetylcholinesterase (AChE) was obtained from PDB with accession number 4PQE. The parameters automatically set by the software are listed as follows: box center, -24-23-0; box size, 20-20-20; random initial conditions, 1; sampling exhaustivity, medium; cavity prioritization, buried. The selection of poses was based on the lowest AC score that was provided as a default by SwissDock.

### 2.3. Computational Chemistry

The Kohn–Sham formulation of density functional theory was employed [21]. The B3LYP was used with the 6-31G(d,p) basis set for all static calculations. All geometry optimizations were carried out using standard convergence criteria and a pruned grid for numerical integration with 99 radial shells and 590 angular points per shell.

The wavefunction stability for each optimized structure was also checked. Solvation effects were considered variationally throughout the optimization procedures via the polarizable continuum model (PCM) [22] using parameters for benzene (or water) and taking advantage of the smooth switching function developed by York and Karplus [23]. Electrophilicity was calculated based on molecular orbital theory [24]. The NBO calculations were carried out as described previously [25].

All the calculations performed in this work were carried out with the Gaussian 09 program.

### 2.4. Experimental Molecular Docking Studies on AChE

Four different X-ray structures that form a stable complex with the co-crystallized ligands/inhibitors (PDB IDs: 4BDT, 4EY5, 4EY6, and 4EY7) were obtained from the RCSB protein database. These four complexes were geometrically optimized by minimization using UCSF-Chimera with the AMBER14SB forced website field [26]. TIP3P [27] was used to explicitly model water and total system charge neutralization with Na^+^Cl^−^ ions. Ligand topologies and parameters were generated with Antechamber [28]. The four optimized complexes were used for cross-docking simulations.

The docking protocol was validated by extracting the co-crystallized ligands from each protein crystal structure and subjecting them to redocking. The accuracy of the docking procedure was evaluated by comparing the predicted binding poses with the experimentally determined crystallographic conformations using the root-mean-square deviation (RMSD). In all cases, the RMSD values obtained from the re-docking were below 2.0 Å: Huprine W (4BDT-ligand), RMSD = 0.710 Å; Huperzine A (4EY5-ligand), RMSD = 0.539 Å; Galanthamine (4EY6-ligand), RMSD = 0.995 Å; 1-benzyl-4-[(5,6-dimethoxy-1-indanon-2-yl)methyl]piperidine (4EY7-ligand), RMSD = 1.675 Å. These results indicate that the docking protocol accurately reproduced the experimentally observed ligand-binding modes; therefore, it was considered suitable for subsequent docking studies.

Ligands were prepared using MolScrub. Relevant tautomers and protonation states at pH = 7.4 were enumerated, and 3D conformers were subsequently generated. Docking simulations were run with GNINA docking software (version 1.3) and results were categorized according to CNN scoring functions [29]. Docking calculations were carried out by setting a grid box according to the dimensions and spatial coordinates of the corresponding co-crystallized ligand for each protein, with an exhaustiveness value of 64 and a maximum output of 20 docking modes. The selection of the final docking poses was based on the CNN affinity scores calculated by GNINA. The poses exhibiting the highest predicted CNN affinities were considered the most favorable binding conformations and were selected for further analysis. Analysis and visual inspection of docking results were carried out using UCSF Chimera (version 1.19) [30]. All ligands were docked to the four optimized complexes. The best docked pose was selected for the most toxic E-isomer.

## 3. Results

### 3.1. Compound Structure and Nomenclature

The list of the compounds that were studied, along with their structures, is presented in Table 1. All compounds exhibit Z- and E-geometric isomers. Mevinphos and VX were included as controls, since they have been extensively studied. The cis-mevinphos corresponds to an E-isomer, while the trans-mevinphos corresponds to a Z-isomer.

In the present study, cis and trans are used to mark the structure of alkyl-substituted cyclohexanol esters in this class of V-agents. One compound, EA 1659 (Table 1), is “locked” in the Z conformation. This is not a chemical warfare agent and was developed as a control compound [4]. It should be noted that all compounds (except mevinphos) display enantiomers at the phosphorus atom. Nevertheless, military-grade nerve agents consist of equal amounts of P_s_ and P_R_ isomers.

### 3.2. Physicochemical and Drug-like Properties

The physicochemical and drug-likeness properties of these compounds were predicted with SwissADME, ADMETlab3, and Deep-PK, and the results are given in Table 2 and Table 3, respectively. Certain features were available for prediction only from one program. For example, density was predicted only by ADMETlab3. For the parameters that were predicted by >1 program, the results are within the same range. Prediction of physicochemical parameters for mevinphos was also carried out as a control, since many of its parameters are experimentally known and have been previously reported.

For all compounds, the numbers of hydrogen-bond acceptors (nHAs), hydrogen-bond donors (nHBs), and rotatable bonds (nRots) are within the optimal range for pharmacologically active molecules (optimal nHAs: 0–12; optimal nHBs: 0–7; optimal nRots: 0–11). The Fsp^3^ is the fraction of sp^3^-hybridized carbons; therefore, the maximum value is 1. Fsp^3^ is an important parameter for drug-likeness, since higher Fsp^3^ values indicate compounds with more complex three-dimensional shapes [31]. Consistently, V-agents exhibit higher values of Fsp^3^ (in the range of 0.75–0.80) relative to mevinphos (0.57).

Regarding the density of the compounds, almost all compounds are predicted to have similar densities (with minor differences), ranging between 1.012 and 1.038. Density is predicted as the MW/van der Waals volume. It should be mentioned that the predictions for density are equal between the E- and Z-isomers. The density of EA 1576 is the only reported experimental value and equals 1.0829 g·mL^−1^ at 25 °C [32], demonstrating good agreement with the prediction. Mevinphos is predicted to have higher density than its phosphonate analogs. Specifically, mevinphos has a density of 1.138 g·mL^−1^, which is also in good agreement with the experimental value of 1.25 g·mL^−1^ at 20 °C (https://pubchem.ncbi.nlm.nih.gov/compound/Mevinphos; accessed on 23 November 2025). The reduction in density from phosphate to phosphonates, as predicted here with mevinphos and its V-type nerve-agent analogs, was previously shown for VG and VE [15], with density reductions from 1.0457 to 1.0180 g·mL^−1^, respectively.

Mevinphos is known to be miscible with water (https://pubchem.ncbi.nlm.nih.gov/compound/Mevinphos; accessed on 23 November 2025) and is predicted to have the highest logS values by all computational tools that were used in this study. The other compounds are also predicted to be soluble in water but with lower solubility than mevinphos. This is in accordance with their chemical structures, since (a) there is a conversion of phosphate to phosphonate ester (removal of one -O- atom) and (b) the compounds contain larger cycloalkyl groups as esters compared to mevinphos. Both these substitutions reduce solubility. Very low variation between the solubility of E- and Z-isomers was predicted.

Compounds exhibiting higher solubility are usually less lipophilic, and indeed, mevinphos has the lowest logP value among its V-type nerve-agent analogs. The V-agents exhibit approximately the same logP values, in accordance with small differences in their chemical formulas (e.g., an addition of a methyl group on the cyclohexyl moiety).

The melting point of E-mevinphos is 21 °C, and that of Z-mevinphos is 6.9 °C. These are very close to the predicted values from Admet3lab of 20.82 °C and 4.944 °C respectively. On the other hand, Deep-PK predicts 9.55 °C and −0.35 °C respectively. The V-agents are predicted to be liquids by both programs, except for EA 1691, which is shown to have a melting temperature of 12.02 °C for the E-isomer and 5.04 °C for the Z-isomer (Deep-PK). The E-isomer of EA 1691 is the only V-agent with an experimentally reported melting point of 43 °C, indicating that EA 1691 is a solid under normal conditions [4]. EA 1576 is also reported to be liquid, without mention of the exact melting point [4]. In conclusion, the predicted melting-point values may differ significantly from the experimental values; therefore, the respective computational tools must be refined.

All compounds exhibited optimal drug-likeness parameters—specifically, the Lipinski filter (MW ≤ 500; logP ≤ 5; nHA ≤ 10; nHD ≤ 5), Ghose filter (160 ≤ MW ≤ 480; −0.4 ≤ WlogP ≤ 5.6; 20 ≤ number of atoms ≤ 70; 40 ≤ molar refractivity ≤ 130), Veber filter (TPSA ≤ 140 Å^2^; nRots ≤ 10); Egan filter (wlogP ≤ 5.88; TPSA ≤ 131.6 Å^2^), and Muegge filter (200 ≤ MW ≤ 600; −2 ≤ xlogP3 ≤ 5; TPSA ≤ 150 Å^2^; number of rings ≤ 7; number of carbons > 4; number of heteroatoms > 1; nHA ≤ 10; nHD ≤ 5) [33]. Furthermore, the compounds follow the Pfizer rule (logP > 3; TPSA < 75 Å^2^) [34], the GSK rule (MW ≤ 400; logP ≤ 4) [35], and the golden triangle rule [200 ≤ MW ≤ 50; −2 ≤ logD ≤ 5 (logP pH 7.4)] [36]. These parameters demonstrate that these compounds are predicted to have favorable ADME profiles, which will be analyzed in more detail below.

### 3.3. Pharmacokinetics/Toxicokinetics

#### 3.3.1. Prediction of Absorption

V-agents are known to mainly act as percutaneous agents due to their low volatility. Therefore, the ability of mevinphos-like V-agents to penetrate the skin was predicted. It was shown that these compounds have higher skin absorption rates than mevinphos, in accordance with their higher lipophilicity. To understand the toxicological impact of the predicted skin Kp values (Table 4), the skin permeability of VX, a well-known percutaneous agent, was also predicted. The permeability of VX was found to be within the range predicted for the mevinphos-like V-agents. Specifically, the Kp for VX was predicted to be 3.55 · 10^−7^ cm·s^−1^ (SwissADME on August 26, 2025) and 1.67 · 10^−7^ cm·s^−1^ (Deep-PK on August 27, 2025). Therefore, the mevinphos-like V-agents are predicted to have high percutaneous absorption. This prediction should be validated in the future.

Furthermore, the potential ability of these V-agents to cross the blood–brain barrier (BBB) and the gastrointestinal tract (GI) was investigated. Regarding the GI, all V-agents show approximately the same values, while all agents are predicted to cross the barrier, except mevinphos (Table 3). This is in accordance with the low lipophilicity of mevinphos that was described above. The permeability of V-agents in the MDCK model shows increased values for mevinphos. Although MDCK permeability is suggested as a potential model for the BBB, since the MDCK cells are canine kidney cells, they may exhibit completely different physiology, which would not allow them to recapitulate the properties of the BBB. Therefore, this prediction warrants future experimental validation.

#### 3.3.2. Prediction of Distribution

For the distribution of mevinphos-type V-agents, the following parameters were predicted: plasma protein binding (PPB), the volume of distribution (VoD), and the unbound fraction in plasma (Fu) (Table 5).

Using ADMET3lab, all V-agents were predicted to show PPB higher than 78%, with mevinphos exhibiting PPB values of 34 and 43% for Z- and E-isomers respectively. This indicates that most mevinphos insecticide is free in plasma, while mevinphos-type V-agents interact with blood plasma proteins, which is expected to lower their active concentration. On the other hand, DeepPK predicted values around 30% for all agents and only approximately 9% for mevinphos. Thus, although the trend is the same, i.e., mevinphos shows lower PPB than its V-type agents, the values deviate a lot, and therefore, no accurate conclusions can be drawn. Both programs predicted very low values of VoD, indicating that most of the mevinphos-type V-agents are found in tissues and not in plasma, which may underline their toxic action. Regarding Fu, low values were predicted by both programs, except for mevinphos, which showed a very large variation.

#### 3.3.3. Prediction of Metabolism

The metabolism of mevinphos-type V-agents by the CYP450 enzymes was predicted, as summarized in Table 6. Specifically, the predictions included classification of nerve agents as substrates or inhibitors of the CYP450 enzymes. SwissADME only provides data for inhibitors of CYP450 and predicted that none of the studied V-agents were inhibitors. Admetlab3 and Deep-PK showed similar results and predicted that mevinphos-type V-agents were not inhibitors or substrates. However, for CYP450 2C19, both tools indicated that the E-isomer is not an inhibitor, while the Z-isomer acts as an inhibitor. In accordance with this finding, compound EA 1659, which is “locked” in the Z-structure, acts as an inhibitor of CYP450 2C19.

Admetlab3 also predicted the potential metabolism of the compounds based on human liver microsomes (HLMs). All compounds were predicted to be metabolized by microsomes (labeled as unstable in Table 6). As a control, VX was predicted to be HLM-unstable (Admetlab 3.0 on 30 September 2025), consistent with what has been experimentally found [37]. Mevinphos is predicted to be stable, i.e., not metabolized by HLM. Indeed, a recent study indicated that when mevinphos is present at a high concentration of 5 mM, it is not metabolized by HLM, while at a very low concentration of 0.5 μM, both E- and Z-isomers are effectively metabolized [38].

#### 3.3.4. Prediction of Excretion

Clearance of the mevinphos-type V-agents was predicted with both ADMETlab3 and DeepPK. The prediction indicated that these agents belong to the class of low- to moderate-clearance agents (low < 5; moderate: between 5 and 15 mL·min^−1^·kg^−1^) (Table 7). The predicted half-life—namely, the time required to reduce the concentration of the V-agent in the plasma to half—indicated these agents are short or ultrashort half-life agents. A large variation in the half-life of mevinphos was observed when predicted with ADMETlab3 and Deep-PK.

### 3.4. Correlation of Toxicity with Structure

E-isomers are more toxic than corresponding Z-isomers. Detailed data have been published for the O,O-diethyl analog of mevinphos, for which the intraperitoneal (i.p.) LD50 in rats is 0.35 mg·kg^−1^ for the E-isomer and 35 mg·kg^−1^ for the Z-isomer, reflecting a 100-fold difference in biological activity [1].

**Figure 1:**
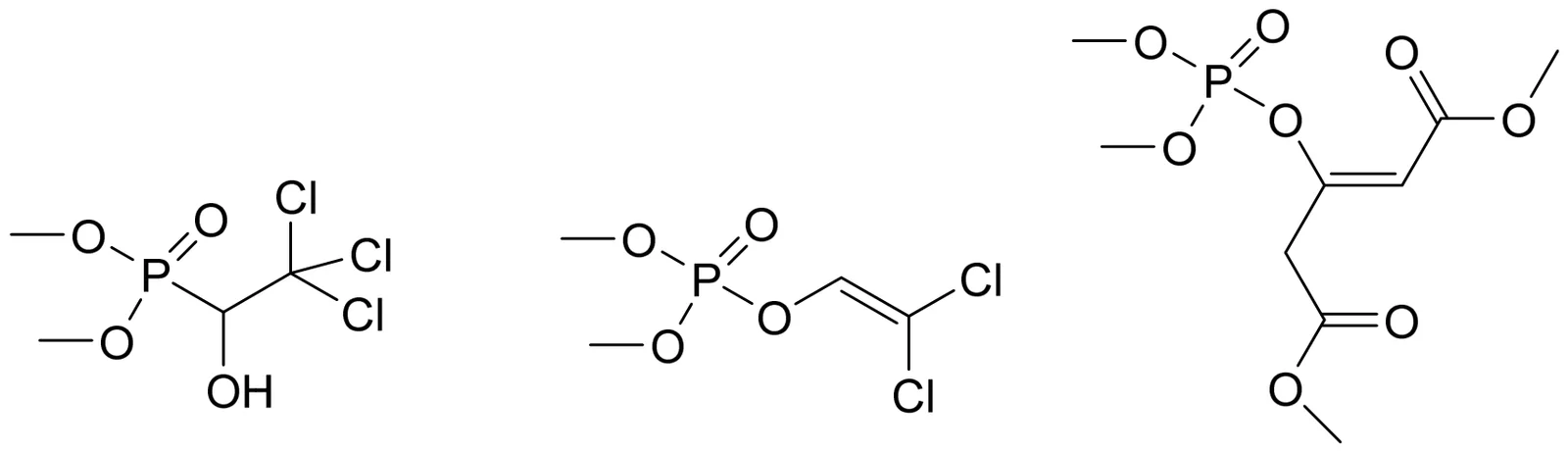
Chemical structures of metrifonate (**left**), dichlorvos (**middle**), and bomyl (**right**).

The presence of electron-withdrawing groups (EWGs) in the leaving group reduces toxicity, as demonstrated with dichlorvos (Figure 1), which is less toxic than mevinphos. This reduced toxicity of dichlorvos is consistent with its use as a drug. Specifically, dichlorvos is the active drug of metrifonate, an organophosphate prodrug for the treatment of Alzheimer’s disease. For comparison, the oral LD50 values in rats are 625 mg·kg^−1^ for metrifonate, 62 mg·kg^−1^ for dichlorvos, and 3.7 mg·kg^−1^ for mevinphos [3].

Replacement of the methyl group on the acetoacetate with an EWG, as shown in bomyl, only slightly increases the toxicity of the E-isomer but severely increases the toxicity of the Z-isomer to a level comparable to that of E (Figure 1) [39].

According to a report by the US Army Chemical Center, EA 1671 and EA 1598 are the most toxic, while EA 1576, which is the carboxy ethyl ester of EA 1598, is less toxic [4]. However, LD50 values were not provided.

**Figure 2:**
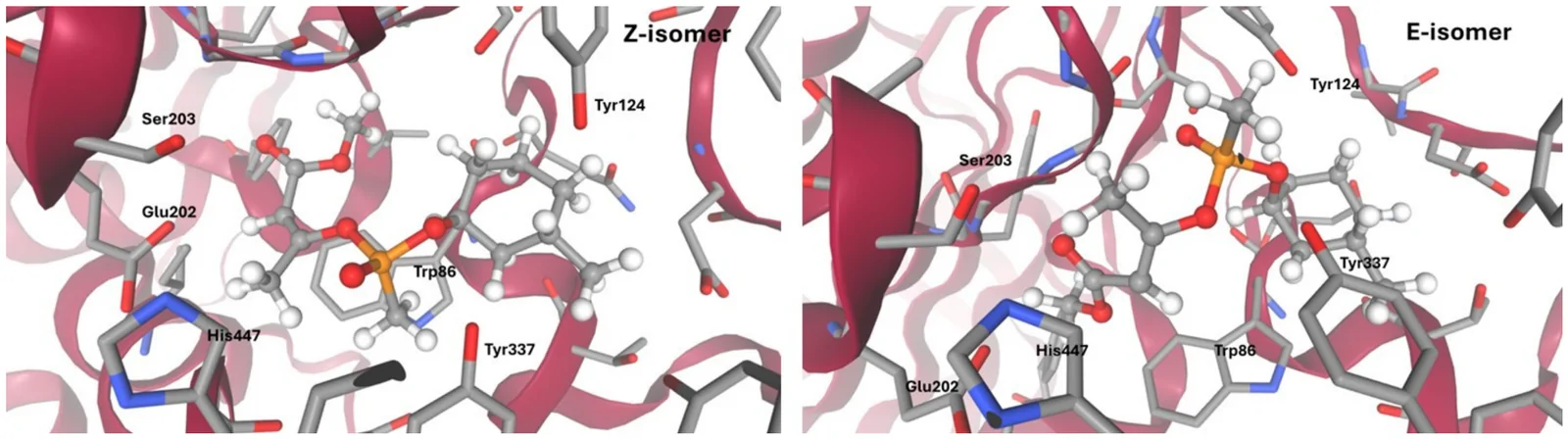
Poses with the lowest energy for the Z- and E-isomers of EA 1598 bound to the active site of AChE as generated by SWISSADME. In both cases, the methylcyclohexyl moiety is in trans orientation. The side chains of the major residues of the active site (including active site Ser203) are marked. Yellow color: phosphorus; gray color: carbon; white color: hydrogen; blue color: nitrogen.

EA 1598 and EA 1576 are 3-methylcyclohexyl phosphonate esters. There is no report on whether the cis- or the trans-isomer of 3-methylcyclohexyl ester is the most toxic. We addressed by performing online docking using Swissdock. Regardless of whether the SMILES formula for the input ligand used for online docking corresponds to E- or Z-isomers, the tool automatically fits both. As a target protein for online docking, the human acetylcholinesterase with PDB accession number 4pqe was used. We inspected the energy of the complex between EA 1598 and AChE. EA 1598 was selected, since it is more toxic than EA 1576. The complex between AChE and EA 1598 exhibited lower energy when either the E- or Z-geometrical isomers had the 3-methylcyclohexyl moiety in trans orientation (the AC scores were −26.315733 and −38.171351, respectively) (Figure 2). Nevertheless, these results should be treated cautiously, since the lowest-energy poses are obtained for the R-enantiomer at phosphorus rather than the S-enantiomer. Indeed, the S-enantiomers on phosphorus of soman [40,41] and VX have been reported to be better inhibitors of AChE with respect to the R-enantiomers [42]. Furthermore, according to recent findings, the S-enantiomers are more toxic than R-enantiomers [40,41,42].

To support our findings, we performed a more detailed docking study. Docking was performed with the human AChE proteins with the following PDB IDs: 4BDT, 4EY5, 4EY6, and 4EY7. The experimental protocol for each enzyme was validated by conducting redocking of the co-crystallized ligands of each protein into their experimentally determined binding sites. The poses were compared with the crystallographic ligand conformations, and the RMSD values were calculated [43]. All RMSD values were below 2.0 Å, confirming the reliability of the docking protocol. The selected derivatives seem to bind to each enzyme in a manner similar to that of the co-crystallized ligands. Protein 4EY5 (which contains huperzine) [44] served as focal point due to the favorable obtained results.

**Figure 3:**
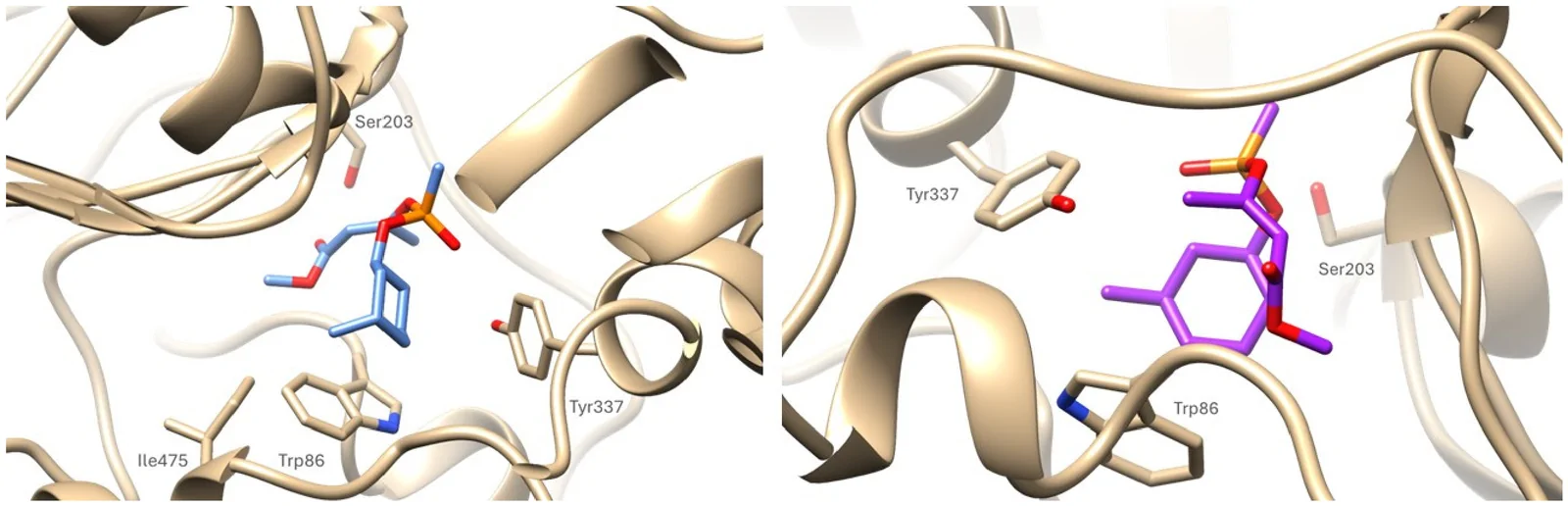
Poses of the E-isomer of EA 1598 with trans-methylcyclohexyl (**Left**) (CNN affinity: 4.957 groups) and cis-methylcyclohexyl (**Right**) (CNN affinity: 4.670 groups).

The E-isomer of EA 1598 containing the trans-methylcyclohexyl group presented better CNN affinity compared to its cis-methylcyclohexyl isomer. Thus, it is predicted to be a better inhibitor. The trans-methylcyclohexyl analog develops hydrophobic interactions with Trp86 and Tyr337, extending into the catalytic anionic site, and Ile451. The cis-methylcyclohexyl analog seems to interact with Trp86 and Tyr337 (Figure 3). Notably, detailed molecular docking analyses showed that EA 1598 binds to AChE with high affinity when an S-enantiomer is present on phosphorus.

At this point, it should be noted that a challenging issue for future studies is the investigation of AChE irreversible inhibition by these agents using a combination of quantum mechanics/molecular mechanics (QM/MM) calculations [45]. Such calculations can also be applied to investigate aging mechanisms, which have important pharmacological implications, as previously reported [46,47].

### 3.5. Stability of E and Z Forms of EA 1671

**Figure 4:**
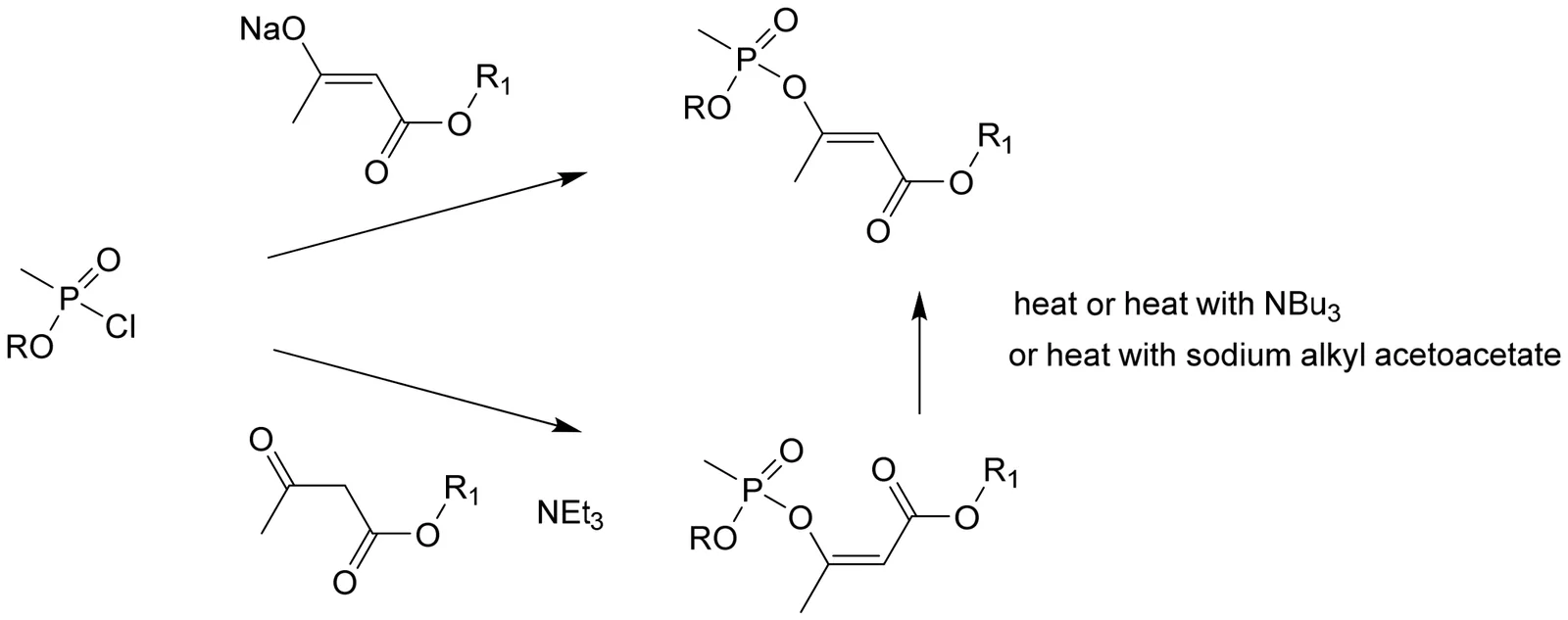
Chemical synthesis of mevinphos-type V-agents. Chemical synthesis can be carried out using two different methods. The first method (**upper part**) involves the reaction of cycloalkyl methylphosphonochloridate with the sodium salt of the alkyl ester of acetoacetate. This yields the E-isomer. The second method (**lower part**) involves the reaction of cyclohexyl methylphosphonochloridate with alkyl ester of acetoacetate in the presence of triethylamine (NEt_3_). This yields the Z-isomer. The Z-isomer can be converted to the more stable E-isomer with heat or a combination of heat and a catalyst.

The chemical synthesis of these compounds is outlined in Figure 4. Briefly, the reaction of sodium salt of alkyl ester of acetoacetates with cyclohexyl methylphosphonochloridate at 80 °C in xylene produces the E-isomer (90% yield for EA 1576). On the other hand, the reaction of alkyl ester of acetoacetate with cyclohexyl methylphosphonochloridate in the presence of triethylamine as a base in benzene produces the Z-isomer (90% yield). The Z-isomer is the labile form and can be converted to E-isomer form with heating alone, or heating with tributylamine, or heating in the presence of a small amount of the respective sodium salt of alkyl ester of acetoacetate. It should be noted that the requirement for increased temperature and, potentially, the presence of a catalyst to convert the Z-isomer to an E-isomer indicates that this conversion cannot occur under physiological conditions. Thus, the reaction with AChE for each isomer is expected to be faster relative to the “inter-conversion” of the isomers.

Using the Gaussian 09 program, the lowest energies for E- and Z-isomers of EA 1671, which is the most toxic compound, were calculated. When the energy calculations were carried out in benzene (the solvent used in chemical synthesis of the Z-isomer), the E-isomer had an energy of −25.3663 kJ·mol^−1^, while the Z-isomer had an energy of −23.8839 kJ·mol^−1^, indicating that the E-isomer exhibits higher stability, in accordance with the experimental data. Nevertheless, when the calculations are carried out in water (the solvent where the biological action takes place) the situation is different, and surprisingly, the Z-isomer appears to have approximately the same energy as the E-isomer (−25.6625 vs. −25.1337 kJ·mol^−1^, respectively).

Future studies with 2D NOESY NMR experiments to monitor the presence of E- and Z-isomers would be highly informative and would facilitate investigation of their stability and potential “inter-conversion”, as has been outlined in other studies [48].

### 3.6. HOMO-LUMO Energies of EA 1671

Then, for EA 1671, the HOMO and LUMO energies, as well as their gap, were calculated as shown in Table 8. LUMO is approximately the same for both isomers, with an energy < 2 eV. A low LUMO energy, as observed here, maximizes the frontier orbital overlap between an electrophile (the V-agent) and the nucleophile (hydroxyl group of Ser203 in AChE or other nucleophiles in a decontamination reagent). This indicates that both E- and Z-isomers of EA 1671 have the tendency to react easily.

### 3.7. Electrophilicity on Phosphorus of Mevinphos-Based V-Type Nerve Agents

To better describe the reactivity, we determined electrophilicity on a phosphorus atom for mevinphos-based V-type nerve agents. VX was also analyzed for comparison. As shown in Table 9, the electrophilicity (ω^+^) is approximately equal for EA 1576, EA 1598, and EA 1671 but approximately 40 times lower than VX, indicating that they are not as reactive towards nucleophiles as the standard VX agent. This includes the reaction towards the active site Ser of AChE.

In addition, we determined the Natural Bond Orbital (NBO). Supplementary Table S1 shows the results of NBO calculation with E2 > 10 kcal·mol^−1^. Compounds EA 1576, EA 1598, and EA 1671 have stronger internal interactions over the phosphorus compared to VX. This finding also supports the notion that although these V-agents are good electrophiles, they are expected to be less reactive than the standard VX agent. Thus, lower reactivity could be related to lower toxicity relative to VX. However, this requires future experimental validation.

## 4. Discussion

Mevinphos-type V-agents have been neither used nor studied extensively. Nevertheless, the potency of these compounds can be understood when studying a case of mevinphos intoxication. Specifically, when a 69 kg person orally took 25 mL of commercial mevinphos, he started to have convulsions and stopped moving within seconds. Death occurred within an estimated 52 s. The pupils were markedly contracted, and the face turned purple. The estimated lethal dose for mevinphos is 280 mg or less than 0.25 mL of the commercial solution, indicating that the person took 100 times more than the lethal dose [49]. There were also two individuals who were accidentally exposed to EA 1671. The first person developed cramps, diarrhea, anxiety, insomnia, and mood changes. The next day, the individual they experienced sweating on the backs of the their hands, and red blood cell (RBC) cholinesterase activity dropped to 11%. The other individual was exposed to EA 1671 while trying to melt the compound and had no symptoms on the first day. The next day, he noted weakness of his eyelids and developed diarrhea. RBC cholinesterase was 15% of normal [50].

The devastating effects of nerve agents have been documented, for example, after the use of sarin in Syria [51,52]; following the use of sarin and VX by Aum Shinrikyo in Japan [53,54]; and with respect to the use of A-agents in assassinations, e.g., that of Alexei Navalny [38] and that of Kim Jong-Nam with VX [55]. These facts have highlighted the need to study their properties with the aim of identifying new decontamination procedures and medical countermeasures.

In this direction, computational approaches allow for the safe study of their properties without the requirement to prepare and handle hazardous materials. Such approaches could lead to more targeted experimental studies for validation in the future, thereby minimizing potential hazards. Computational methods have been used to study the physicochemical and toxicological properties of G- and A-type nerve agents [56], and molecular dynamics have been applied to understand the physicochemical properties of G-agents [57]. Quantum chemical calculations have also been carried out for the theoretical analysis of the stability of mevinphos in the environment [58].

Here, we applied computational tools to study the metabolism of drugs [16,59]. For the most part, online programs were used to characterize the toxicokinetic profiling of the mevinphos-type V-agents. All V-agents were predicted to have optimal medicinal chemistry properties, i.e., drug-likeness compatible with specific bioactive compounds, and optimal physicochemical properties. Regarding ADME, most predictions indicate that these compounds exhibit optimal pharmacokinetic profiling.

AChE has a good nucleophilic OH group from active site Ser 203, which can readily react with good electrophiles. It was calculated that both E- and Z-isomers have very low LUMO energies (<2 eV), indicating that they are very electrophilic and can readily react. The higher toxicity that is associated with an increased ability to inhibit AChE is probably favored for the E-isomer for stereochemical reasons and owing to its better ability to interact with amino-acid side chains within the active site pocket. We also found that trans-methylcyclohexyl derivatives are more toxic than the cis-derivatives, meriting detailed experimental work in the future. There are also other freely available software programs that apply empirical valence-bond approaches to study the chemical kinetics of enzymes, the (bio)chemical reactivity [60,61,62] of which could be applied in future studies as well.

The mevinphos-type V-agents also bear a cyclohexyl moiety instead of a simple alkyl group, e.g., methyl or ethyl. This is related to increased lipophilicity, which, in turn, is related to increased predicted skin permeability. Furthermore, this alkyl group is expected to remain attached to the phosphonylated AChE. It is known that bulky groups offer increased resistance to classical oxime reactivation due to steric hindrance. This has been considered to account for the greater resistance to oxime reactivation of AChE inhibited by cyclosarin (GF) relative to VR and VR relative to VX, since cyclosarin generates a cyclohexyl methylphosphonate adduct (as the compounds described here); VR, an isobutyl methylphosphonate adduct; and VX, an ethyl methylphosphonate adduct [63]. Based on this fact, it can be speculated that the methylcyclohexyl V-agents would be more resistant to oxime reactivation than the standard VX or VR agents.

The importance of E- and Z-geometry with respect to toxicity is demonstrated by the fact that the E-isomer of EA 1576 was reported to be ten times more toxic than the Z-isomer, although the exact LD50 values were not provided [4]. The difference between the E- and Z-isomer toxicity of the diethyl analog of mevinphos is 100-fold [1]. The conversion of phosphate (mevinphos) to phosphonate (V-agent) may result in highly toxic compounds that compensate for their relative E/Z-ratio toxicity. Attempts to predict the toxicity of mevinphos-based V-agents were not carried out with Web-based software, since, as demonstrated previously, they result in erroneous results [64]. Certain mevinphos-like V-agents include esters of 3-methylcyclohexanol. Whether the 3-methylcyclohexyl group is in cis or trans orientation was not reported [4,5]. Here, we used molecular docking to predict which of the two isomers could bind preferentially to the active site of AChE. Our analyses showed that the trans-isomer is the best. This requires future validation experiments due to the potential limitations of molecular docking [65,66]. This is also important given the fact that SwissDock showed docking with the R-enantiomer, while our more detailed analyses showed docking with the S-enantiomer.

Computational methods eliminate the need for physical samples and laboratory equipment, thereby reducing the cost significantly. In toxicological studies, they reduce the number of experimental animals required and potential ethical concerns. However, there is a training-data bias, meaning that they provide more accurate data for chemicals used in their training dataset. Furthermore, they may offer limited or poor accuracy for novel chemical scaffolds. This fact was previously highlighted by the inability of commercial ADMET programs to predict the LD50s of V-agents. The differences in accuracy of SwissADME, ADMETlab3.0, and pkCSM in terms of CYP-mediated metabolism have been a matter of a very recent discussion [67]. Therefore, there is a need for standardization of validation criteria. Although computational methods provide predictions that require experimental validation, they can significantly reduce the number of experiments by suggesting specific/targeted experiments and can narrow down the questions that need to be addressed. Indeed, FDA and EMA regard in silico predictions as supplementary evidence for drug development.

At this point, it should be noted that although these agents are irreversible inhibitors of AChE (as nerve agents in general), reversible inhibitors of AChE are important from a clinical perspective, since they constitute a main class of drugs used in Alzheimer’s disease [68,69]. Recently, irreversible inhibitors of AChE have been suggested as new drugs to improve the beneficial effects on Alzheimer disease [70]. It may be interesting for future studies to change the structure of these agents with the aim of reducing toxicity and generating new pharmacological agents for AChE. Indeed, similar studies resulted in the generation of glaucoma drug echothiophate (phospholine iodide), which shares similarities with the VG agent [71].

In conclusion, our computational study justifies the further investigation of this unexplored class of V-agents. The data provided here will assist in the design of future targeted experiments to delineate the biological activity of this unexplored class of V-agents, especially given the concerns of potential chemical terrorism and the availability of procedures to synthesize these compounds. Additional future studies with more advanced modelling are also required to shed light on the effect of phosphorus stereochemistry in binding to the active site of AChE. Finally, it should be noted that this is an in silico study, and all toxicological conclusions need to be verified in future experimental studies.

## Figures and Tables

**Table 1:** Chemical structure and simplified molecular-input line-entry system (SMILES) of the V-agents and pesticides studied here.

Compound	Chemical Structure	SMILES
Mevinphos (E-isomer)	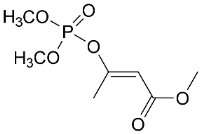	COC(=O)\C=C(/C)OP(=O)(OC)OC
Mevinphos (Z-isomer)	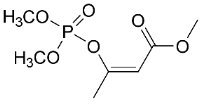	COC(=O)\C=C(\C)OP(=O)(OC)OC
EA 1576 (E-isomer)	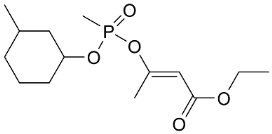	CCOC(=O)\C=C(/C)OP(C)(=O)OC1CCCC(C)C1
EA 1576 (Z-isomer)	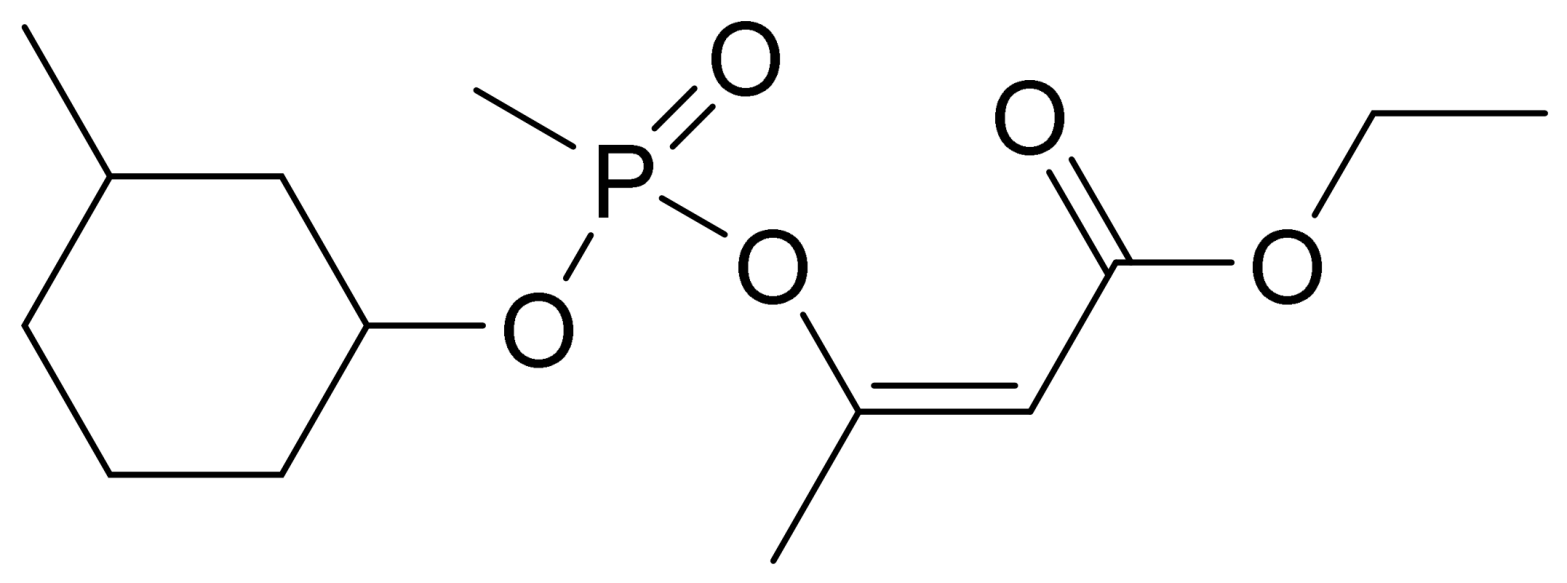	CCOC(=O)\C=C(\C)OP(C)(=O)OC1CCCC(C)C1
EA 1598 (E-isomer)	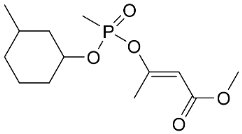	COC(=O)\C=C(/C)OP(C)(=O)OC1CCCC(C)C1
EA 1598 (Z-isomer)	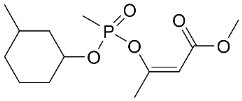	COC(=O)\C=C(\C)OP(C)(=O)OC1CCCC(C)C1
EA 1671 (E-isomer)	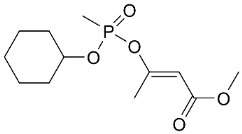	COC(=O)\C=C(/C)OP(C)(=O)OC1CCCCC1
EA 1671 (Z-isomer)	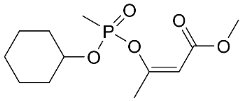	COC(=O)\C=C(\C)OP(C)(=O)OC1CCCCC1
EA 1659 (Locked)	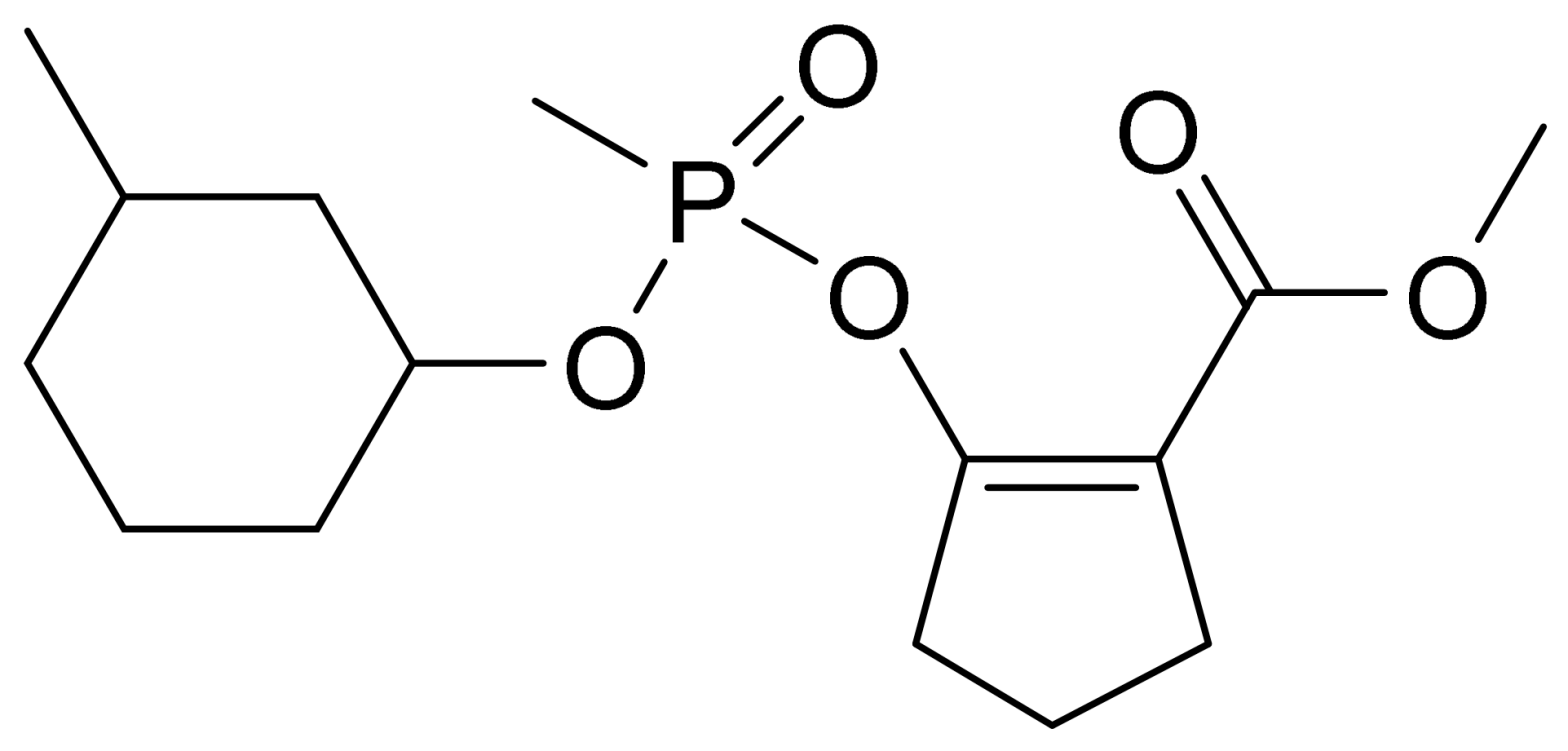	COC(=O)C1=C(CCC1)OP(C)(=O)OC1CCCC(C)C1
EA 1701 (VX)	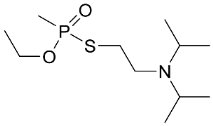	CCOP(C)(=O)SCCN(C(C)C)C(C)C

**Table 2:** Physicochemical properties of mevinphos-like V-agents.

	EA 1576			EA 1598			EA 1671			EA 1659			Mevinphos		
	SA *	A3 *	DPK *	SA	A3	DPK	SA	A3	DPK	SA	A3	DPK	SA	A3	DPK
MW	304.32	304.14	304.32	290.29	290.13	290.27	276.27	276.11	276.27	316.33	316.14	316.33	224.15	224.04	224.15
vW volume		300.649			283.353			266.057			309.389			196.924	
nHA	5	5	5	5	5	5	5	5	5	5	5	5	6	6	6
nHD	0	0	0	0	0	0	0	0	0	0	0	0	0	0	0
nRot	7	7	7	6	6	6	6	6	6	6	6	6	6	6	6
TSPA	78.94	61.83	61.83	71.64	61.83		71.64	61.83	61.83	71.64	61.83	61.83	80.87	71.06	71.06
Fsp^3^	0.79	0.79		0.77	0.77		0.75	0.75		0.80	0.80		0.57	0.57	
logS, mol·l^−1^ (E) **	−2.97	−2.574	−3.05	−2.72	−2.035	−2.58	−2.36	−1.829	−2.15	−3.07	−2.182	−3.0	−1.59	−0.004	−0.20
logS, mol·l^−1^ (Z)	−2.97	−2.235	−3.01	−2.72	−1.874	−2.5	−2.36	−1.725	−2.07				−1.59	−0.272	−0.12
logP (E)	2.74	2.844	3.2	2.42	2.147	2.69	2.16	1.977	2.31	2.78	2.09	2.89	0.99	0.263	0.88
LogP (Z)	2.69	2.408	2.99	2.36	2.04	2.48	2.14	1.826	2.11				0.98	0.187	0.66
Density, g·mL^−1^ (E)		1.012			1.024			1.038			1.022			1.138	
Density, g·mL^−1^ (Z)		1.012			1.024			1.038						1.138	
m.p., °C (E)		−32.849	−11.0		−18.689	3.2		−18.82	12.02		−11.155	2.31		20.82	9.55
m.p., °C (Z)		−36.969	−19.27		−22.522	−4.78		−24.925	5.04					4.944	−0.35

* SA, SwissADME; A3, ADMETLAB3; DPK, DeepPK; ** E and Z indicate the E- and Z-isomers, respectively; MW: molecular weight; vW: van der Waals; nHA number of hydrogen-bond acceptors; nHD number of hydrogen-bond donors; nRot, number of rotatable bonds; TSPA: Topological Surface Polar Area; logS: logarithm of aqueous solubility; logP: logarithm of n-octanol/water distribution; m.p. melting point.

**Table 3:** Medicinal chemistry properties of mevinphos-like V-agents.

	EA 1576		EA 1598		EA 1671		EA 1659		Mevinphos	
Filter	SA	A3	SA	A3	SA	A3	SA	A3	SA	A3
Lipinski	Yes	Yes	Yes	Yes	Yes	Yes	Yes	Yes	Yes	Yes
Pfizer		Yes		Yes		Yes		Yes		Yes
GSK		Yes		Yes		Yes		Yes		Yes
Golden triangle		Yes		Yes		Yes		Yes		Yes
Veber	Yes		Yes		Yes		Yes		Yes	
Ghose	Yes		Yes		Yes		Yes		Yes	
Egan	Yes		Yes		Yes		Yes		Yes	
Muegge	Yes		Yes		Yes		Yes		Yes	

**Table 4:** Prediction of absorption for mevinphos-like V-agents.

	EA 1576				EA 1598				EA 1671				EA 1659				Mevinphos			
	SA	A3	DPK *	Con **	SA	A3	DPK	Con	SA	A3	DPK	Con	SA	A3	DPK	Con	SA	A3	DPK	Con
BBB	Yes				Yes				Yes				Yes				No			
GI	Yes				Yes				Yes				Yes				Yes			
Caco-2, 10^−5^ cm·s^−1^ (E)		1.58	3.09	2.34		1.81	3.09	2.45		2.81	3.09	2.95		2.43	2.81	2.62		1.62	1.26	1.44
Caco-2, 10^−5^ cm·s^−1^ (Z)		1.28	3.09	2.19		1.16	3.09	2.13		1.91	3.09	2.50						1.50	1.26	1.38
MDCK, 10^−5^ cm·s^−1^ (E)		1.38	4.57	2.98		1.5	5.37	3.44		2.06	6.92	4.49		1.44	3.98	2.71		2.39	17.8	N.D. #
MDCK, 10^−5^ cm·s^−1^ (Z)		1.24	4.57	2.91		0.98	5.37	3.18		1.77	6.92	4.35						1.89	17.8	N.D.
Skin *K*p, 10^−7^ cm·s^−1^	5.9		3.34	4.62	3.89		3.05	3.47	2.34		2.48	2.41	4.37		2.78	3.58	1.51		1.56	1.54

* Only predictions in the training range are shown for Deep-PK; ** Con, consensus calculated as median; # N.D., not determined (due to large variation); BBB: blood–brain barrier; GI, gastrointestinal tract.

**Table 5:** Prediction of distribution properties of mevinphos-like V-agents.

	EA 1576		EA 1598		EA 1671		EA 1659		Mevinphos	
	A3	DPK	A3	DPK	A3	DPK	A3	DPK	A3	DPK
PPB (%) (E)	97.26	31	90.99	26.28	82.93	25.91	91.12	35.84	43.40	9.24
PPB (%) (Z)	91.77	31.03	87.52	26.29	78.11	25.83			34.18	9.67
VoD (l) (E)	0.85	2.75	0.63	3.09	0.38	3.47	0.53	2.04	0.49	0.28
VoD (l) (Z)	0.81	2.82	0.48	3.09	0.32	3.47			0.47	0.28
Fu (%) (E)	3.39	3.89	8.20	3.80	15.17	3.55	10.94	4.90	48.35	0.87
Fu (%) (Z)	7.28	3.89	13.26	3.80	19.31	3.55			59.59	0.87

PPB: plasma protein binding; VoD: volume of distribution; Fu: unbound fraction in plasma.

**Table 6:** Prediction of metabolism of mevinphos-like V-agents by CYP450 enzymes and human liver microsomes (HLM).

	EA 1576			EA 1598			EA 1671			EA 1659			Mevinphos		
CYP450	SA	A3	DPK	SA	A3	DPK	SA	A3	DPK	SA	A3	DPK	SA	A3	DPK
1A2 inhibitor	No	No	Yes	No	No	No	No	No	No	No	No	Yes	No	Yes E No Z	Yes
1A2 substrate		No	No		No	No		No	No		No	No		No	No
2C19 inhibitor	No	No E * Yes Z	No E Yes Z	No	No	No E Yes Z		No	No E Yes Z	No	Yes	Yes	No	Yes	Yes
2C19 substrate		No E Yes Z	No		No	No		No	No		No	No		No E Yes Z	No
2C9 inhibitor	No	No	Yes	No	No	Yes	No	No	No	No	No	Yes	No	No	No
2C9 substrate		No	Yes		No (E) Yes (Z)	Yes		No	Yes		No	Yes		Yes	No
2D6 inhibitor	No	No	No	No	No	No	No	No	No	No	No	No	No	No	No
2D6 substrate		No	No		No	No		No	No		No	No		Yes	No
3A4 inhibitor	No	No	No	No	No	No	No	No	No	No	Yes	No	No	No	No
3A4 substrate		No	No		No	No		No	No		No	No		No	No
2B6 inhibitor		Yes			No			Yes			No			Yes	
2B6 substrate		No			No			No			No			Yes	
2C8 inhibitor		Yes			Yes			Yes			Yes			Yes E No Z	
HLM stability		Unstable			Unstable			Unstable E Stable Z			Unstable			Stable	

* E and Z are only labeled when they are predicted to exhibit different properties. In all other cases, E- and Z-isomers are predicted to have identical metabolism.

**Table 7:** Prediction of excretion of mevinphos-based V-type nerve agents.

	EA 1576		EA 1598		EA 1671		EA 1659		Mevinphos	
	A3	DPK	A3	DPK	A3	DPK	A3	DPK	A3	DPK
CL_plasma_, mL·min^−1^·kg^−1^ (E)	5.399	4.8	6.219	3.77	5.158	2.99	6.487	4.31	8.732	6.3
CL_plasma_, mL·min^−1^·kg^−1^ (Z)	5.765	4.84	6.694	3.8	5.605	3.03			8.82	6.29
T_1/2_, hours (E)	0.688	<3	0.846	<3	0.868	<3	0.725	<3	1.284	≥3
T_1/2_, hours (Z)	0.704	<3	0.813	<3	0.844	<3			1.161	≥3

**Table 8:** Calculation of the HOMO-LUMO energies for compound EA 1671.

	EA 1671 E-Isomer	EA 1671 Z-Isomer
HOMO (eV)	−6.824	−6.897
LUMO (eV)	−1.010	−1.027
E gap = LUMO-HOMO (eV)	5.814	5.870

**Table 9:** Calculation of electrophilicity on a phosphorus atom (calculation in water).

	HOMO *	LUMO *	μ **	n ***	ω = μ^2^/2n	NPA (P)(N)	NPA(P)(N + 1)	f_k_^+^ = q^N+1^-q^N^	ω^+^ = f_k_^+^ω
VX	−0.20482	0.00517	−0.10241	0.20999	0.024972	2.05355	1.8724	0.18115	0.0045237
ΕA 1576 Ε	−0.25524	−0.04591	−0.12762	0.20933	0.038902	2.43158	2.42814	0.00344	0.0001338
ΕA 1598 Ε	−0.25662	−0.04724	−0.12831	0.20938	0.039315	2.43065	2.42792	0.00273	0.0001073
ΕA 1671 Ε	−0.25593	−0.04674	−0.127965	0.20919	0.039139	2.43128	2.42808	0.0032	0.0001252

* Hartree units; ** μ = (HOMO + LUMO)/2; *** n = LUMO-HOMO. The calculations were performed only for E-isomers (the more toxic isomers).

## Data Availability

All data are included in the manuscript.

## Supplementary Materials

The following supporting information can be downloaded at: https://www.mdpi.com/article/10.3390/cimb48080779/s1.

## Dual Use Research Statement

All data within the manuscript are computational predictions, and there are no experimental hazards involved, since no synthesis or handling of these agents was carried out. This study reports the chemical and toxicological predictions of an unexplored class of V-agents with the aim of highlighting the potential dangers and concerns that may be derived from their illegal use. This work aims only to contribute to public safety, and the outcomes are solely be used for peaceful applications. For this reason, certain details of their chemical synthesis have been omitted.

## Abbreviations

The following abbreviations are used in this manuscript:

ADME	Absorption–Distribution–Metabolism–Excretion
HLM	Human Liver Microsome
HOMO	Highest Occupied Molecular Orbital
LUMO	Lowest Occupied Molecular Orbital
RMSD	Root-Mean-Square Deviation
