# Supplementary material for: Computational Chemistry and Toxicology of Phosphonate Esters of Alkyl Acetoacetates, an Unexplored Class of V-Agents
Source: Curr Issues Mol Biol. 2026 Jul 30;48(8):779. doi: 10.3390/cimb48080779 (PMC13510841; doi:10.3390/cimb48080779)
Supplement: Supplementary file 1 [file cimb-48-00779-s001.zip › cimb-4413816-supplementary.pdf]

*Supplementary Materials*

# Computational Chemistry and Toxicology of Phosphonate Esters of Alkyl Acetoacetates, an Unexplored Class of V-Agents

**Georgios Pampalakis [1,*] and Eleni Pontiki [2]**

[1] Laboratory of Pharmacology, School of Pharmacy, Aristotle University of Thessaloniki, 54124 Thessaloniki, Greece
[2] Laboratory of Pharmaceutical Chemistry, School of Pharmacy, Aristotle University of Thessaloniki, 54124 Thessaloniki, Greece; epontiki@pharm.auth.gr
* Correspondence: gpampalakis@pharm.auth.gr

**Table S1.** Calculation of electrophilicity on phosphorus atom (calculation in water).

| V-agent | Donor | Acceptor | E2 (kcal·mol$^{-1}$) |
|---|---|---|---|
| VX | LP(2) O3 | BD*(1) P1-S4 | 12.53 |
| | LP(2) O3 | BD*(1) P1-C5 | 16.47 |
| | LP(3) O3 | BD*(1) P1-O2 | 22.42 |
| | LP(3) O3 | BD*(1) P1-S4 | 12.91 |
| EA 1576 E-isomer | BD(1)P1-O2 | BD*(2)P1-O3 | 45.61 |
| | BD(1)P1-O2 | BD*(1)P1-O4 | 21.92 |
| | BD(1)P1-O3 | BD*(2)P1-O3 | 18.19 |
| | BD(1)P1-O3 | BD*(1)P1-O4 | 19.63 |
| | BD(2)P1-O3 | BD*(1)P1-O2 | 17.01 |
| | BD(2)P1-O3 | BD*(1)P1-O4 | 15.47 |
| | BD(1)P1-O4 | BD*(1)P1-O2 | 25.75 |
| | BD(1)P1-O4 | BD*(1)P1-O3 | 15.93 |
| | BD(1)P1-O4 | BD*(2)P1-O3 | 37.88 |
| | LP(1) O2 | BD*(1)P1-O3 | 12.09 |
| | LP(1) O3 | BD*(1)P1-C5 | 16.82 |
| EA 1598 E-isomer | BD(1)P1-O2 | BD*(2)P1-O3 | 45.42 |
| | BD(1)P1-O2 | BD*(1)P1-O4 | 21.75 |
| | BD(1)P1-O3 | BD*(2)P1-O3 | 18.67 |
| | BD(1)P1-O3 | BD*(1)P1-O4 | 19.61 |
| | BD(2)P1-O3 | BD*(1)P1-O2 | 16.96 |
| | BD(2)P1-O3 | BD*(1)P1-O3 | 10.45 |
| | BD(2)P1-O3 | BD*(1)P1-O4 | 15.23 |
| | BD(1)P1-O4 | BD*(1)P1-O2 | 25.58 |
| | BD(1)P1-O4 | BD*(1)P1-O3 | 16.20 |
| | BD(1)P1-O4 | BD*(2)P1-O3 | 37.01 |
| | LP(1) O2 | BD*(1)P1-O3 | 12.17 |
| | LP(1) O3 | BD*(1)P1-C5 | 16.85 |
| EA 1671 E-isomer | BD(1)P1-O2 | BD*(2)P1-O3 | 45.22 |
| | BD(1)P1-O2 | BD*(1)P1-O4 | 21.82 |
| | BD(1)P1-O3 | BD*(2)P1-O3 | 18.54 |
| | BD(1)P1-O3 | BD*(1)P1-O4 | 19.88 |
| | BD(2)P1-O3 | BD*(1)P1-O2 | 16.89 |
| | BD(2)P1-O3 | BD*(1)P1-O3 | 10.04 |
| | BD(2)P1-O3 | BD*(1)P1-O4 | 15.40 |
| | BD(1)P1-O4 | BD*(1)P1-O2 | 25.67 |
| | BD(1)P1-O4 | BD*(1)P1-O3 | 16.23 |
| | BD(1)P1-O4 | BD*(2)P1-O3 | 37.65 |
| | LP(1) O2 | BD*(1)P1-O3 | 12.07 |
| | LP(1) O3 | BD*(1)P1-C5 | 16.82 |
